# Hypertrophic pachymeningitis associated with myelodysplastic syndrome

**DOI:** 10.1002/jha2.68

**Published:** 2020-08-04

**Authors:** Akane Kaihatsu, Kyoko Fuse, Hirohito Sone, Masayoshi Masuko

**Affiliations:** ^1^ Department of Hematology Endocrinology and Metabolism Niigata University Faculty of Medicine Niigata Japan; ^2^ Department of Hematopoietic Cell Transplantation Niigata University Medical and Dental Hospital Niigata Japan

A 62‐year‐old male was diagnosed with myelodysplastic syndrome‐multi‐lineage dysplasia (MDS‐MLD), pericarditis, and generalized erythema due to autoimmune disorders associated with MDS. Although one course of azacytidine therapy and prednisolone (0.5 mg/kg/day) improved his symptoms, the patient declined further treatment.

Seven months later, he developed severe headache and deafness. Subsequently, he had nose obstruction and facial nerve palsy. In addition, he gradually developed dysphagia and eye movement limitation. Cranial magnetic resonance imaging (MRI) revealed thickened dura mater, which was enhanced with gadolinium (Figure 1, T1 fast field echo method, white arrow; thickened dura mater, black arrow; no thickening of the dura mater). Lumbar puncture demonstrated no evidence of infection or malignant tumor. He was thus diagnosed with hypertrophic pachymeningitis (HP) associated with MDS. Intravenous methylprednisolone pulse (1 g/day for 3 days) followed by prednisolone at 2 mg/kg/day was ineffective. He declined further azacytidine therapy and died from aspiration pneumoniae.

**FIGURE 1:**
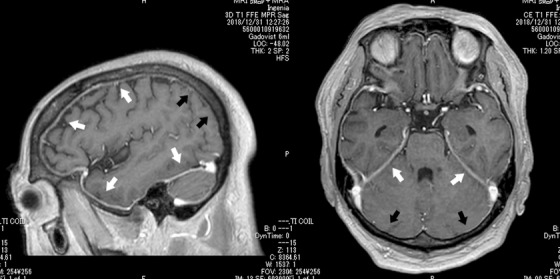
Cranial MRI (T1 fast field echo method, enhanced with gadolinium)

HP is classified as idiopathic or secondary from infection, autoimmunity, or malignant tumor. Chronic inflammation causes thickening of the dura mater, and the pathology consists of fibrosis of the dura mater and lymphocyte infiltration. HP causes neurological symptoms, headache, hearing loss, facial paralysis, and cerebellar ataxia. HP usually is diagnosed by MRI.

Ten percent to 28% of MDS patients have concomitant autoimmune disease such as polyarthritis, collagen disease, vasculitis, Sweet disease, inflammatory bowel disease, and acute pericarditis. Fraison et al. (Leuk Res. 2016) reported that 87% of 22 patients who used azacytidine for MDS‐related autoimmune disease had improved symptoms, and 63% were able to reduce steroid dose [1].

Although no therapy for HP associated with MDS has been established, rapid diagnosis and azacytidine therapy may improve the outcome.

## CONFLICT OF INTEREST

The authors have no conflicts of interest directly relevant to the content of this article.
